# Impact of remote monitoring on well-being, therapeutic adherence, and organ damage evaluation in hypertensive patients: the PROSIT study

**DOI:** 10.1093/ehjdh/ztag001

**Published:** 2026-01-06

**Authors:** Marialuisa S Marozzi, Vanessa Desantis, Francesco Corvasce, Giuseppe S Falcone, Marilena Santovito, Gianmarino Colleoni, Monica Montagnani, Angelo Vacca, Sebastiano Cicco

**Affiliations:** Unit of Internal Medicine ‘Guido Baccelli’, Department of Precision and Regenerative Medicine and Ionian Area- (DiMePRe-J), University of Bari Aldo Moro, AUOC Policlinico, Piazza Giulio Cesare, 11, Bari 70124, Italy; Unit of Hypertension ‘A.M. Pirrelli’, Department of Precision and Regenerative Medicine and Ionian Area – (DiMePRe-J), University of Bari Aldo Moro, AUOC Policlinico, Bari, Italy; Unit of Pharmacology, Department of Precision and Regenerative Medicine and Ionian Area – (DiMePRe-J), University of Bari Aldo Moro, Bari, Italy; Unit of Internal Medicine ‘Guido Baccelli’, Department of Precision and Regenerative Medicine and Ionian Area- (DiMePRe-J), University of Bari Aldo Moro, AUOC Policlinico, Piazza Giulio Cesare, 11, Bari 70124, Italy; Unit of Hypertension ‘A.M. Pirrelli’, Department of Precision and Regenerative Medicine and Ionian Area – (DiMePRe-J), University of Bari Aldo Moro, AUOC Policlinico, Bari, Italy; Unit of Internal Medicine ‘Guido Baccelli’, Department of Precision and Regenerative Medicine and Ionian Area- (DiMePRe-J), University of Bari Aldo Moro, AUOC Policlinico, Piazza Giulio Cesare, 11, Bari 70124, Italy; Exprivia SpA, Molfetta, Bari, Italy; STMicroelectronics Srl, Unit of Lecce, Lecce, Italy; Unit of Pharmacology, Department of Precision and Regenerative Medicine and Ionian Area – (DiMePRe-J), University of Bari Aldo Moro, Bari, Italy; Unit of Internal Medicine ‘Guido Baccelli’, Department of Precision and Regenerative Medicine and Ionian Area- (DiMePRe-J), University of Bari Aldo Moro, AUOC Policlinico, Piazza Giulio Cesare, 11, Bari 70124, Italy; Unit of Internal Medicine ‘Guido Baccelli’, Department of Precision and Regenerative Medicine and Ionian Area- (DiMePRe-J), University of Bari Aldo Moro, AUOC Policlinico, Piazza Giulio Cesare, 11, Bari 70124, Italy; Unit of Hypertension ‘A.M. Pirrelli’, Department of Precision and Regenerative Medicine and Ionian Area – (DiMePRe-J), University of Bari Aldo Moro, AUOC Policlinico, Bari, Italy

**Keywords:** Telemedicine, Remote patient monitoring, Arterial hypertension, Hypertension-mediated organ damage, e-Health

## Abstract

**Aims:**

The PROSIT (Patient-Reported Outcomes and Smart-Imaging in Telecardiology) study aimed to evaluate the feasibility and potential clinical impact of remote patient monitoring in hypertension management, focusing on medication adherence, therapeutic optimization, and organ damage.

**Methods and results:**

We conducted a prospective single-center, randomized pilot study involving 100 hypertensive Caucasian patients, assigned to three groups: Group A, equipped with a wearable ECG device, and a mobile application reporting vital signs; Group B, using only the mobile app; and Group C (control), standard care. Blood pressure and heart rate were measured with validated devices. Medication adherence was assessed with the validated Medication Adherence Report Scale (MARS-5). Echocardiography and biochemical parameters were evaluated at baseline and after 12 months, including a 6-month washout period without digital support. Patients in Groups A and B showed significantly higher MARS-5 scores, vs. Group C (*P* = 0.001). Early therapeutic adjustments were more frequent in Groups A and B, leading to a reduction in the number of prescribed antihypertensive medications (median decrease from 2 [2–3] to 1 [0–3], *P* = 0.001). At follow-up, Group A exhibited a significant reduction in interventricular septum thickness and left ventricular mass (*P* = 0.01) along with improved renal function (A *P* = 0.04, B *P* = 0.02).

**Conclusion:**

This study suggests that telemedicine with remote monitoring may enhance medication adherence and allow early treatment optimization with fewer drugs, accompanied by favorable changes in cardiac and renal parameters. These findings warrant confirmation in larger, multicenter studies.

## Graphical Abstract

**Graphical Abstract:**
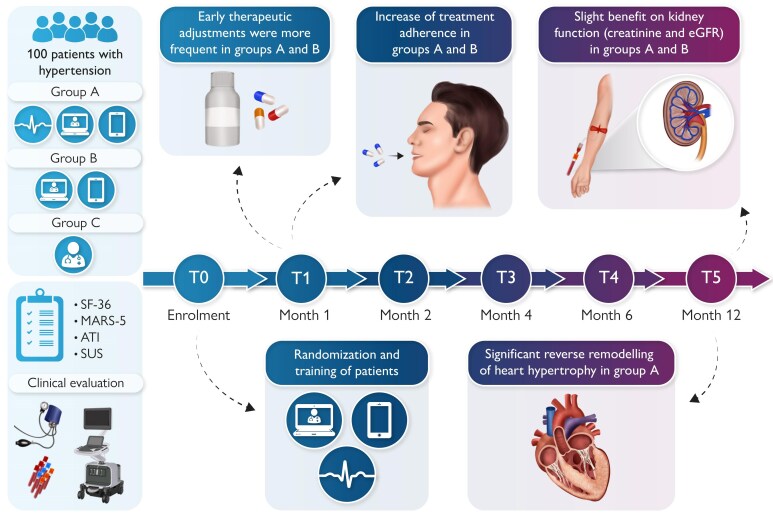

## Introduction

Hypertension is a leading risk factor for cardiovascular diseases (CVD), including stroke, coronary artery disease, heart failure, and chronic kidney disease.^1^ The correlation between blood pressure levels (both systolic and diastolic) and CVD remains significant regardless of other risk factors. Notably, hypertensive individuals tend to experience CVD approximately 5 years earlier than normotensive ones.^2^ The negative impact of arterial hypertension and its complications on patients’ quality of life (QoL) is significant.^3^ Various factors can be involved, including age, education, gender, duration of the disease, number of medications taken, and systolic pressure values.^4,5^ The flawed QoL also correlates with significant organ damage mediated by hypertension (HMOD). Conversely, Germanova *et al*. found that hypertensive patients with severe HMOD showed a significant impairment in mental and physical health, particularly concerning daily activities.^6^

In this context, educational and behavioral strategies are increasingly needed to promote patient participation in disease management and adherence to therapy. Importantly, these strategies must consider the socio-economic background and the healthcare settings in which patients are managed. Greater attention to self-management in chronic disease is often a necessary step with extremely positive effects on clinical outcomes and QoL.^7^ Enhancing self-management in chronic conditions has been shown to improve both clinical outcomes and perceived QoL. Telemedicine and, more generally, e-Health may play a key role in this regard. By leveraging digital health technologies, e-Health enables the intelligent processing of clinical data, facilitating personalized interventions, continuous monitoring, and improved patient care.^8^

This study was designed as a pilot evaluation to assess the feasibility and usability of a multilevel digital intervention in a real-world cohort of treated, clinically stable hypertensive patients. The primary aim of this study was to explore the association between telemonitoring, medication adherence, and QoL of hypertensive patients and to examine their potential relationship with surrogate markers of organ function. A secondary aim was to evaluate patient compliance with the digital system and identify potential barriers to its broader implementation in routine care.

## Patients and methods

### Study design and protocol

We carried on a single-center, prospective, randomized controlled trial. Patients were enrolled at the time of their first visit (baseline, T0), and subsequently assessed at 1 (T1), 2 (T2), 4 (T3), and 6 (T4) months. At baseline, complete clinical evaluation including medical and treatment history was recorded. At this time point, routine laboratory tests to evaluate kidney function and cardio-metabolic risk (Plasma LDL, HDL, total cholesterol, triglycerides, and glucose levels) were measured. According to international guidelines,^9,10^ a complete echocardiographic evaluation was performed. All ultrasounds have been performed by a single expert sonographer, using the same ultrasound system (Canon Aplio i700 Imaging System), with a 1.5–3 MHz probe (Canon Medical Systems Europe B.V., Amsterdam, The Netherlands). The sonographer was blinded to the study group and to the previous measurements at the time of follow-up evaluations. During the scheduled follow-ups, disease progression and treatment efficacy were monitored by clinical evaluation and questionnaire assessment. Patients equipped with telemedicine systems (Groups A and B) underwent continuous remote monitoring, regardless of scheduled visits, allowing for real-time data collection and early detection of potential issues. In the six months after the end of follow-up (washout), no patient performed any control, either clinical or using telemonitoring system. At the end of the washout (six months without the possibility of using telemonitoring and/or mobile app), patients were re-evaluated in an additional follow-up visit (T5) exploring clinical status and routine laboratory parameters to further investigate long-term clinical outcomes, disease progression, and treatment adherence. At T5 echocardiogram was repeated by the same physician with the same instrument used at T0. This final evaluation was intended to provide insights into the sustained effects of the intervention and the overall health status of the participants. Changes in antihypertensive therapy were evaluated by recording the number and class of medications at different timepoints. The study was conducted according to the Declaration of Helsinki and approved by the Comitato Etico Territoriale Azienda Ospedaliero-Universitaria Consorziale Policlinico di Bari, Bari, Italy (protocol n. 319, March 30, 2023). Patients signed a written informed consent before enrollment.

Inclusion criteria: arterial hypertension (defined as SBP ≥140 mmHg and/or DBP ≥90 mmHg or current use of antihypertensive therapy); age ≥ 18 years old; ownership of a smartphone or mobile device with compatible operating system capable of running the study application and ensuring regular access to mobile data or Wi-Fi connectivity.

Exclusion criteria: absence of arterial hypertension; inability to participate due to functional limitations (e.g. severe cognitive impairment, physical disability preventing use of the intervention or adherence to the protocol) ; age ≤ 18 years old; lack of ownership or access to compatible mobile device.

### Study population

We evaluated 100 Caucasian patients diagnosed with arterial hypertension, randomized into three groups based on key clinical characteristics (age, gender, left ventricular ejection fraction, presence of atrial fibrillation, and pulmonary embolism), to ensure balance distribution across the groups at baseline:

- **Group A (*n***  **=**  **34, 19 males/15 females, aged 60.79**  **±**  **13.12 years):** patients were equipped with a wearable device (Hi) for continuous monitoring of electrocardiography and a dedicated mobile app for manual input of vital signs.
- **Group B (*n***  **=**  **33, 21 males/12 females, aged 62.12**  **±**  **12.93 years):** patients equipped only with the mobile app for self-reporting vital signs.
- **Group C (*n***  **=**  **36, 21 males/15 females, aged 61.64**  **±**  **13.88 years):** patients receiving standard care, with no access to telemonitoring or app-based self-management (Control group).

The app used by patients in Groups A and B was designed to suggest patient-physician interactions based on the input data.

### Web app and telemonitoring

The telemonitoring system was based on a secure, cloud-based software solution, designed and implemented by Exprivia SpA to facilitate remote patient management and improve the monitoring of hypertension. The system included a mobile app application that allowed patients to report their vital signs manually, including blood pressure, heart rate, and other relevant clinical parameters, as part of their routine health data input, view trend graphs, receive educational content, and communicate with healthcare providers. The system aimed to facilitate patient participation in self-monitoring and to improve the management of hypertension by physicians. The app also sent reminders for measurements and displayed feedback based on thresholds defined in the care pathway.

The telemonitoring system enabled continuous tracking of vital signs, allowing for early identification of technological or clinical issues. Blood pressure and heart rate were measured at home using validated automatic upper-arm sphygmomanometers listed in the STRIDE BP database. Patients were instructed to manually enter these values into the mobile app at least twice weekly or upon significant symptoms. Additionally, the decision support system deployed in the platform along with the Electronic medical record (EMR) component assessed the entered data against a predefined diagnostic therapeutic care pathway (DTCP) and automatically validated the patient's condition in real-time. It supported clinical decision-making by suggesting therapy adjustments, lifestyle recommendations, or specialist referral based on patient data trends. The system generated alerts for healthcare providers if any critical threshold was reached, supporting timely intervention and specific care. Alerts were generated when predefined thresholds were exceeded and were reviewed by a dedicated physician within 24 h. The DTCPs were developed in accordance with the 2023 ESH Guidelines and Italian national telemedicine protocols. The system was also designed to facilitate follow-up discussions through a teleconsultation feature, enabling healthcare providers to schedule, monitor, and perform virtual visits as part of the ongoing management of the patient’s condition. Patients in Groups A and B received scheduled teleconsultations, at T2 and T4, and if asked via app, in addition to on-demand contact triggered by alerts, for clinical urgent reasons (only one patient asked for it).

The cloud-based nature of the system ensured that all data were securely stored, and privacy and security accomplished; moreover, data were easily accessible for healthcare providers, enabling the remote monitoring of patients between visits. All data entered into the app were encrypted and transmitted to a secure cloud platform, accessible via a clinical web interface. The system enabled structured and traceable interactions between patients and the care team. All clinical actions and contacts were automatically logged in the system, although these were not quantitatively analyzed in this study. All actions triggered by abnormal findings were logged, and patient safety was ensured through monitoring and prompt escalation of critical cases. This approach aimed to improve the management of hypertension and other cardiovascular conditions by promoting adherence to treatment, and providing real-time clinical insights to guide decision-making.

The wearable device used was a 3 lead ECG, a non-invasive system utilized for remote cardiovascular monitoring, provided by STMicroelectronics Srl. The device provided continuous rhythm monitoring (at least 30 min twice weekly or as needed), chosen to detect arrhythmic events that may accompany hypertension.

### Training and education

All healthcare professionals involved in the patient management underwent a structured training program. This training included theoretical and practical sessions on the use of telemonitoring devices, data interpretation, and patient support strategies to ensure standardized care delivery and consistent data collection across all study participants.

On the day of enrollment patients in Groups A and B received a dedicated personal training session. This training explained the correct use of the wearable device (for Group A), the mobile application functionalities, and the proper procedure for self-reporting vital signs. The procedure was explained at enrollment and periodically reviewed during follow-up to ensure correct data input. In the event of any difficulties, patients had the possibility to contact the care team by sending a message directly through the application. The goal was to ensure correct data entry and promote adherence to the telemonitoring protocol.

### Questionnaires administered

The following validated questionnaires were employed:

1. SF-36 (Short Form Health Survey—36 items): the SF-36 is a widely used questionnaire for evaluating health-related QoL. It consists of 36 questions divided into eight domains: physical function, role limitations due to physical health problems, bodily pain, general health perceptions, vitality, social functioning, role limitations due to emotional problems and mental health. Scores range from 0 to 100, with higher values indicating better QoL. It was administered at the beginning of the study (T0) and during subsequent follow-ups to monitor the evolution of the analyzed parameters.^11^
2. ATI (Affinity for Technology Interaction): the ATI questionnaire measures the degree of technology acceptance by users. This tool assesses an individual's predisposition to interact with digital technologies and electronic devices, providing insight into patients’ adaptability to telemonitoring systems. It was administered at the beginning of the study (T0).^12^
3. SUS (System Usability Scale): the SUS is a standardized questionnaire to evaluate the usability of a digital system, in this case, the web app used for remote monitoring. It consists of 10 statements rated on a 5-point Likert scale, scored on a 0–100 scale. The final score provides an indication of user experience, where a value above 68 is considered indicative of a system with good usability. It was administered at the end of the study.^13^
4. MARS-5 (Medication Adherence Report Scale—5 items): this questionnaire assesses medication adherence. It is a validated self-report questionnaire and consists of five questions that investigate patients’ behavior in following their prescribed treatment regimen. The total score ranges from 5 to 25, with higher scores indicating greater adherence to treatment. It was administered to patients at the beginning of the study (T0) and during subsequent follow-ups to monitor the evolution of the analyzed parameters.^14^

### Statistical analysis

The collected data were analyzed using GraphPad Prism software (La Jolla, CA, USA), and expressed as mean ± standard deviation (SD) for parametric variables and as median and interquartile range (IQR) for non-parametric variables. The Kolmogorov–Smirnov test was used to assess the normal distribution of the data. For non-normally distributed variables, the Mann–Whitney test was used for comparisons, and Spearman's rank correlation was used for correlations. For normally distributed data, the parametric *t*-test for unpaired data was used for comparisons, and Pearson's correlation was used for correlations. The Chi-square test was used for the distribution of dichotomous variables. The evaluation of different time points compared to T0 within the same group was performed using Friedman analysis and subsequent Wilcoxon signed-rank test for non-parametric data, while for parametric data, the paired *t*-test was performed after evaluation using ANOVA. A *P*-value <0.05 was considered to indicate a statistically significant difference. In order to evaluate our aim, we consider α = 0.05, β = 0.1, values and standard deviation found in previously.^15^ With these parameters, a sample size of 100 patients was deemed sufficient for the objectives of this prospective randomized study. Therefore, we included at least 33 patients to each group.

## Results

### Basal characteristics

Patients in all groups were comparable for comorbidities distribution (see Supplementary material online, *Table S1*). Also, drugs administered in terms of number or type of medication assumed at T0 were similar between the three groups (see Supplementary material online, *Table S2*). Few statistically significant differences between groups were observed in both clinical and laboratory parameters at T0 (see Supplementary material online, *Table S3*), as well as in echocardiographic parameters (see Supplementary material online, *Table S4*). The only significances, not clinically relevant, were LDL cholesterol (B vs. C *P* = 0.040)(see Supplementary material online, *Table S3*) and about echocardiographic parameters E velocity (A vs. B *P* = 0.046), septal interventricular wall (A vs. C *P* = 0.031), left ventricular mass (g) (A vs. C *P* = 0.021) and left ventricular mass index (g/m^2^)(A vs. C *P* = 0.014)(see Supplementary material online, *Table S4*).

### Questionnaires

Based on the predefined DTCP, the software generated notifications for physicians, providing therapeutic recommendations or alert codes when necessary. All notifications were found to be consistent with the clinical data reported by patients, ensuring the reliability of the decision support system.

Among the characteristics of the application, the presence of multiple features was the most appreciated (by approximately 50% of patients), followed by the speed of interactions, preferred by 19% of patients. Analysis of the ATI questionnaires at the time of enrollment revealed no statistically significant difference between the answers provided by patients of the Groups A, B, and C (see Supplementary material online, *Figure S1 A*). However, a significant positive correlation emerged when the educational background (both in terms of years of education and type of diploma) was correlated with the patient’s affinity for technology interaction (see Supplementary material online, *Figure S1C*-*D*). This was particularly evident for individuals with higher education levels (up to 18 years of schooling) (see Supplementary material online, *Figure S1C*) or holding advanced degrees (see Supplementary material online, *Figure S1D*). This indicates that higher digital literacy favored better adaptability to telemonitoring systems, suggesting the need for targeted educational strategies in technologically less experienced populations. Similarly, there was no difference in SUS scale evaluation between Group A and B patients at the end of the study (see Supplementary material online, *Figure S1B*).

**Figure 1:**
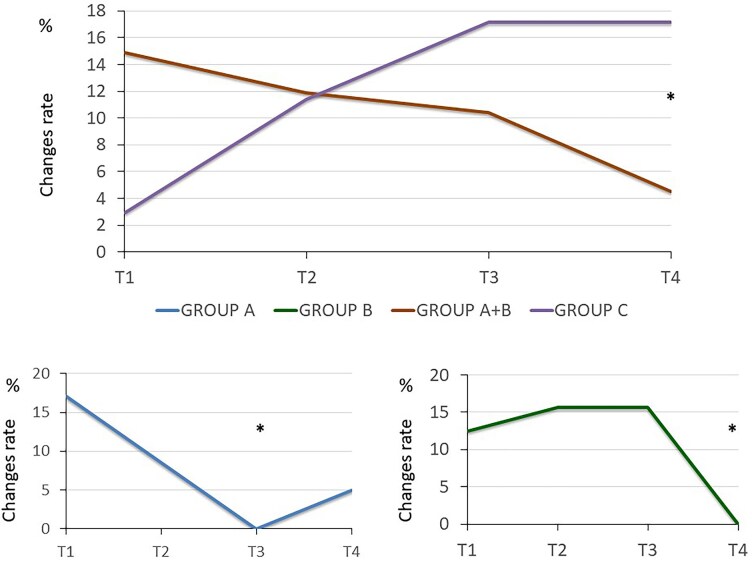
Percentage of patients showing a therapeutic change in Groups A, B, and C at the experimental time points. The aggregated data of A + B is also considered, showing that the reduction in therapeutic changes remains constant up to T4. (*) indicates the statistical significance (*P* < 0.05) between the three groups at the time point indicated.

The SF-36 questionnaire showed no statistically significant improvements in any domain among Groups A or B compared with baseline, while a modest decline in emotional well-being was observed in the control group (Group C).

### Follow-up analysis:

Medication adherence, assessed with the validated MARS-5 questionnaire, significantly increased in all groups at T1 compared with baseline (A and B *P* = 0.001; C *P* = 0.01), remaining stable throughout the study and after washout (*Figure 3*; Supplementary material online, *Table S6*). The improvement was more pronounced in Groups A and B than in controls at every timepoint (*P* = 0.001). Patients in the two intervention groups achieved comparable adherence trajectories, except for a slight difference at T4 (Group B vs. Group A, *P* = 0.001).

Early therapeutic adjustments were more frequent in Groups A (17.14% at T1) and B (12.5% at T1), whereas late changes were predominant in the control group (17.14% at T4) (*Figure 1*).

**Figure 2:**
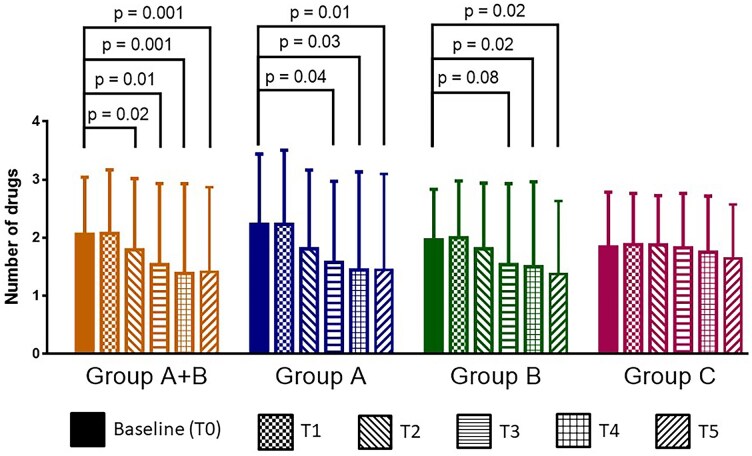
Number of medications taken and the significance of the differences found between Groups A (blu bars), B (green bars), and C (purple bars) at the experimental time points. The aggregated data of A + B are also considered (yellow bars).

A significant reduction in the number of prescribed antihypertensive medications was observed in Groups A and B (median decrease from 2 [2–3] to 1 [0–3] at T4 and T5) (*Figure 2*; Supplementary material online, *Table S7*). In contrast, dosage escalations were sometimes required for patients from Group C throughout the study period, and the total number of prescribed drugs remained unchanged in this group (*Figures 1* and *2*, Supplementary material online, *Table S7*).

**Figure 3:**
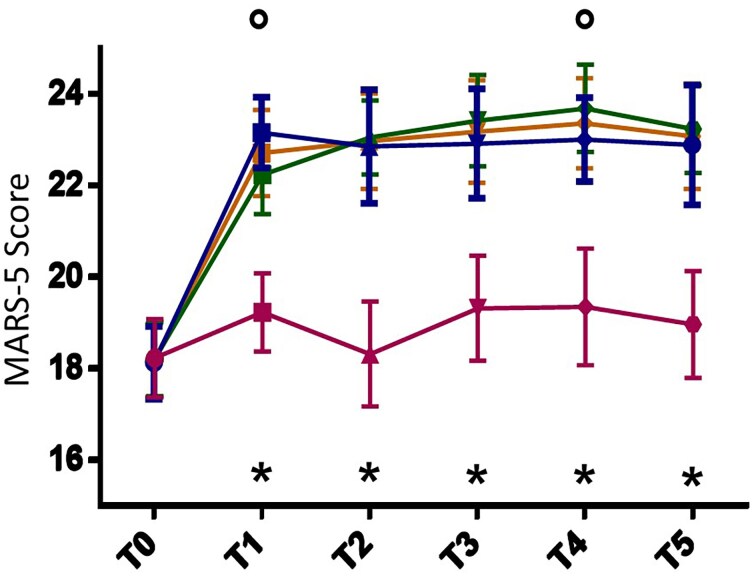
Treatment adherence evaluated using MARS-5 score differences found between Groups A (blue line), B (green line), and C (purple line) at the experimental time points. The aggregated data of A + B are also considered (yellow line). (°) indicates the statistical significant (*P* < 0.05) difference comparing Group A and B. (*) indicates the statistical significant (*P* < 0.001) difference comparing Group C and both Groups A and B.

### Echocardiographic and renal findings

**Figure 4:**
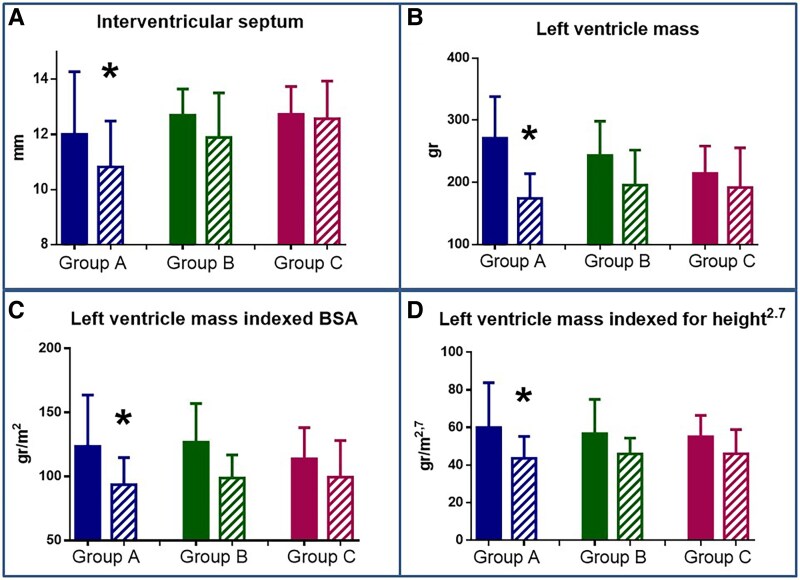
Changes at 1-year (striped bars) echocardiography evaluation compared to baseline (plain bars) of hypertension-mediated organ damage in each group of interventricular septum (panel A), absolute left ventricle mass (panel B) and left ventricle mass indexed for body surface area (BSA—panel C) and for height elevated to 2.7 (Panel D). (*) indicates the statistical significance (*P* < 0.05) between baseline and 1-year follow-up.

At the 12-month follow-up (including six months of washout), routine instrumental assessments revealed a significant reverse heart remodeling only in patients from the Group A. In particular, patients in Group A showed significant reductions in interventricular septal thickness (*P* = 0.01), LV mass (*P* = 0.01), and LV mass indexed for body surface area (BSA) and height²·⁷ (*P* = 0.03) compared with baseline (*Figure 4*; *Table 1*). No significant echocardiographic changes were detected in Groups B or C.

**Table 1:** Comparison of echocardiographic parameters at baseline (T0) and at T5 in patients from Groups A, B, and C

	Group A			Group B			Group C		
	Baseline (T0)	1 year (T5)	*P*	Baseline (T0)	1 year (T5)	*P*	Baseline (T0)	1 year (T5)	*P*
**Left ventricle ejection fraction (%)**	61.79 ± 7.44	59.36 ± 3.63	0.159	62.72 ± 3.55	61.60 ± 4.53	0.789	61.31 ± 4.00	60.31 ± 4.77	0.307
**Septal interventricular wall (mm)**	13.61 ± 1.50	10.82 ± 1.62	**0**.**010**	12.92 ± 1.08	11.89 ± 1.62	0.195	12.73 ± 0.99	12.56 ± 1.37	0.726
**Posterior wall diameter**	13.00 ± 1.57	10.36 ± 2.01	**0**.**020**	12.68 ± 1.25	12.11 ± 2.80	0.386	12.133 ± 1.48	12.27 ± 2.37	0.502
**Internal diameter of the left ventricle (mm)**	49.22 ± 5.13	47.18 ± 2.79	0.172	47.57 ± 4.83	45.11 ± 3.89	0.417	47.045 ± 4.53	44.60 ± 4.69	0.081
**Left ventricular mass (g)**	271.16 ± 66.98	174.50 ± 38.67	**0**.**010**	243.07 ± 55.27	195.60 ± 56.50	0.474	224.89 ± 53.07	191.80 ± 63.77	0.606
**Left ventricular mass index (g/m^2^)**	141.77 ± 31.72	93.73 ± 20.49	**0**.**010**	126.76 ± 30.26	98.85 ± 17.91	0.425	118.50 ± 24.2	99.48 ± 28.54	0.443
**Left ventricular mass index^2.7^ (g/m^2.7^)**	69.04 ± 19.45	43.66 ± 11.31	**0**.**030**	60.55 ± 14.45	45.94 ± 8.44	0.462	59.05 ± 14.16	46.05 ± 12.91	0.332
**Relative wall thickness**	0.53 ± 0.07	0.43 ± 0.08	**0**.**020**	0.55 ± 0.07	0.51 ± 0.09	0.729	0.52 ± 0.08	0.53 ± 0.07	0.249
***E* velocity (cm/s)**	69.55 ± 18.03	75.29 ± 21.85	0.440	67.47 ± 15.94	74.14 ± 8.82	0.815	66.33 ± 15.49	68.00 ± 14.87	0.609
***A* velocity (cm/s)**	83.26 ± 18.35	64.19.14	**0**.**040**	72.33 ± 13.21	67.14 ± 14.78	0.951	78.83 ± 18.55	71.30 ± 13.47	0.225
***e′* velocity (cm/s)**	7.31 ± 2.00	9.14 ± 2.74	0.356	8.21 ± 2.20	9.00 ± 1.73	0.423	8.07 ± 2.14	9.33 ± 3.88	0.752
***E/e′***	9.86 ± 3.47	8.86 ± 2.51	0.412	8.42 ± 1.17	8.67 ± 1.52	0.798	8.65 ± 1.86	8.00 ± 2.5	0.698

Renal function improved in both intervention groups, with decreased serum creatinine (A *P* = 0.04; B *P* = 0.02) and increased estimated glomerular filtration rate (A *P* = 0.03; B *P* = 0.04) (*Table 2*).

**Table 2:** Comparison of laboratory parameters at baseline (T0) and at T5 in patients from Groups A, B, and C

	Group A			Group B			Group C		
	Baseline (T0)	1 year (T5)	*P*	Baseline (T0)	1 year (T5)	*P*	Baseline (T0)	1 year (T5)	*P*
**Creatinine (mg/dL)**	0.94 ± 0.29	0.82 ± 0.21	**0**.**040**	0.96 ± 0.30	0.88 ± 0.13	**0**.**020**	0.88 ± 0.23	0.92 ± 0.18	0.503
**Glomerular filtration rate (mL/min)**	80.83 ± 19.65	86.67 ± 19.67	**0**.**030**	81.23 ± 21.38	87.33 ± 16.33	**0**.**040**	86.15 ± 20.48	82.00 ± 17.64	0.842
**Total cholesterol (mg/dL)**	168.5 ± 34.73	143.50 ± 33.50	0.201	174.28 ± 38.35	167.50 ± 36.25	0.207	158.15 ± 31.28	162.10 ± 34.07	0.777
**LDL cholesterol (mg/dL)**	100.91 ± 34.18	74.83 ± 33.71	0.068	109.96 ± 34.98	88.50 ± 31.92	0.154	89.58 ± 27.40	89.14 ± 28.60	0.380
**HDL cholesterol (mg/dL)**	50.77 ± 12.42	48.50 ± 8.57	0.946	52.29 ± 13.04	56.63 ± 16.27	0.945	52.42 ± 16.32	49.00 ± 13.03	0.710
**Triglycerides (mg/dL)**	104.18 ± 40.01	95.83 ± 36.36	0.785	90.417 ± 28.39	95.00 ± 41.19	0.208	103.72 ± 32.35	114.90 ± 45.31	0.716
**Glucose (mg/dL)**	97.87 ± 24.92	94.83 ± 42.13	0.887	92.88 ± 17.04	94.13 ± 12.60	0.107	90.71 ± 11.82	106.20 ± 25.03	**0**.**030**
**Urinary protein to creatinine ratio**	133.09 ± 66.34	73.00 ± 100.60	0.290	128.83 ± 134.17	91.88 ± 80.08	0.088	120.67 ± 167.20	94.86 ± 125.10	0.089
**Urinary albumin to creatinine ratio**	20.43 ± 11.50	14.50 ± 15.55	0.210	25.91 ± 85.85	33.25 ± 76.40	0.395	7.50 ± 4.89	6.28 ± 2.83	0.635

In contrast, Group C showed a mild worsening in fasting glucose at T5 (*P* = 0.03).

### Blood pressure and heart rate

Office blood pressure values were comparable at baseline among the three groups. A small but statistically significant reduction in diastolic BP was observed in Group B at T3 (*P* = 0.04), while heart rate changes across groups were minor and clinically irrelevant (see Supplementary material online, *Figure S2*).

Home BP readings were not analyzed statistically due to variable frequency and number of patient-reported entries.

### Quality of life

The SF-36 questionnaire did not reveal significant improvements in any domain for Groups A or B, whereas a slight decline in emotional well-being was observed in Group C. ANOVA identified overall group differences but no specific pairwise contrasts. These data indicate that the telemonitoring intervention did not significantly influence self-reported QoL.

In summary, telemonitored patients (Groups A and B) demonstrated greater medication adherence, earlier therapeutic optimization, and a reduced pharmacological burden compared with standard care. These changes were accompanied by favorable trends in renal function and cardiac remodeling, while blood pressure values and QoL remained largely stable.

## Discussion

The integration of digital technologies, such as remote patient monitoring, offers opportunities to improve chronic disease management and support individualized care. However, while remote monitoring shows benefits for selected conditions, evidence on its long-term effectiveness and cost-efficiency remains limited, requiring further investigation for wider clinical implementation.^16^ The PROSIT study leverages wearable technology and patient-reported data to evaluate the feasibility and potential clinical effects of telemonitoring in hypertension. Given the clinically stable profile of the enrolled population, the aim was not to evaluate treatment efficacy, but to examine how digital tools may influence therapeutic adherence, medication optimization, and surrogate markers of organ function in a real-world setting. The main finding of this study was a significant improvement in medication adherence among telemonitored patients, as assessed by MARS-5, accompanied by a reduction in the number of prescribed antihypertensive drugs. This suggests that remote monitoring may enhance patient awareness and communication with healthcare providers, leading to earlier and more targeted therapeutic adjustments. Similar results have been reported in previous telemonitoring studies, where improved adherence was associated with better disease control and treatment continuity.^17,18^ The present findings also highlight favorable changes in cardiac and renal parameters, including a reduction in LV mass and improved eGFR in telemonitored groups. These data may reflect more stable blood pressure control and greater adherence to treatment, although the magnitude of LV mass regression should be interpreted cautiously. Given the normotensive status and medication reduction observed in this cohort, physiological mechanisms alone are unlikely to explain such changes. Therefore, this result is considered hypothesis-generating and may partly reflect behavioral factors or measurement variability, including the Hawthorne effect. QoL, assessed by the SF-36 questionnaire, remained overall stable, with no significant improvement in the intervention groups and a modest decline in the control group. This indicates that telemonitoring primarily affected adherence and surrogate outcomes, without measurable changes in perceived well-being during the study period.

Barriers to telemonitoring adoption were also identified. Some patients reported technical difficulties or limited digital literacy, which required support from the clinical team. These findings are consistent with the literature, where usability and familiarity with technology are key determinants of long-term adherence and participation.^19–24^ Tailored educational and technical support programs remain essential to maximize engagement and ensure equitable access to digital healthcare.

From a clinical perspective, telemonitoring provides new means to better track the patient’s health: by taking advantage of an online platform, healthcare personnel can evaluate patients (even through teleconsultations), and monitor their clinical conditions via a real electronic health record, even in rural communities or in elder subjects.^25,26^ This is particularly important in a chronic setting, where periodic review of patients’ parameters can be crucial as well as tools to alert healthcare personnel in case of an acute event. In the context of hypertension, where long-term monitoring is essential, timely intervention is particularly beneficial. This could help reduce hospitalizations, optimize therapeutic strategies, and enhance patient safety by allowing rapid adjustments in treatment protocols. Concomitantly, the constant access to clinical and anamnestic data of the subjects can help identify potential risk categories where monitoring is even more crucial. However, no definitive conclusions on clinical efficacy or long-term cardiovascular outcomes can be drawn from this pilot study.

The observation of early therapeutic adjustments in telemonitored patients suggests that closer follow-up may enable clinicians to optimize therapy before deterioration occurs. These results underscore the potential of telemonitoring to complement traditional care, improving adherence and clinical coordination while reducing pharmacological burden.^27–29^ To our knowledge, this is the first study that assessed the key role of telemedicine strategies in potentially contribute reducing hypertension-mediated organ damage. In fact, the reverse remodeling of the heart and the improved kidney function strengthen the role of remote monitoring allowing a more effective hypertension control. In summary, the PROSIT study suggests that remote monitoring may strengthen medication adherence, support early therapy optimization, and positively influence cardiac and renal surrogate markers in hypertensive patients.These findings provide preliminary evidence of the clinical value of integrating telemonitoring into standard care, warranting confirmation in larger, multicenter studies with extended follow-up and cost-effectiveness evaluation.

### Limitation

This study presents some limitations that should be acknowledged. First, 24-hour ambulatory blood pressure monitoring (ABPM) was not included, which may limit the precision of blood pressure trend evaluation. Second, medication adherence was assessed through the validated MARS-5 questionnaire, although no biochemical confirmation was available. The observed changes in left ventricular mass and renal function, particularly in Group A, should be interpreted with caution. The observed improvements may partly reflect a Hawthorne effect, whereby participants modified their behavior because of increased observation and interaction. Despite these limitations, the consistency of the adherence and organ function findings suggests a meaningful trend that warrants further confirmation.

## Conclusion

In conclusion, this pilot study suggests that telemonitoring may help improve medication adherence and support early therapeutic optimization in patients with well-controlled hypertension. The digital intervention was associated with a reduction in the number of prescribed antihypertensive drugs and favorable changes in cardiac and renal parameters, although these results should be interpreted with caution. Behavioral factors, including the Hawthorne effect, may have contributed to the observed improvements.

By integrating remote monitoring into standard follow-up, clinicians may obtain additional, objective information on treatment adherence and early response to therapy. Such an approach could promote more personalized management of hypertension without increasing medication burden.

Further multicenter studies with larger populations and longer observation periods are required to confirm these preliminary findings and to assess their cost-effectiveness, reproducibility, and long-term clinical impact. A more robust evidence base will be essential to determine the role of telemonitoring as a complementary tool in the routine care of hypertensive patients.

## Supplementary Material

ztag001_Supplementary_Data

## Data Availability

The data are not publicly available due to ethical restrictions.
