# Supplementary material for: Impact of remote monitoring on well-being, therapeutic adherence, and organ damage evaluation in hypertensive patients: the PROSIT study
Source: Eur Heart J Digit Health. 2026 Jan 6;7(2):ztag001. doi: 10.1093/ehjdh/ztag001 (PMC12835817; doi:10.1093/ehjdh/ztag001)
Supplement: ztag001_Supplementary_Data [file ztag001_supplementary_data.docx]

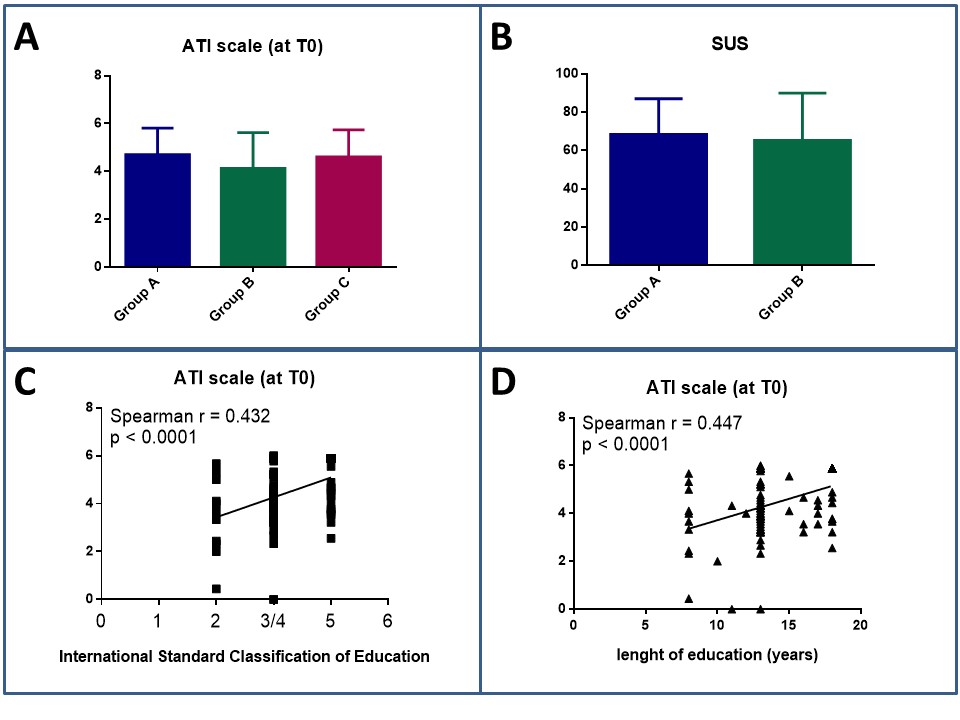


**Supplementary Figure 1.** The affinity for technology interaction (ATI) at T0 (Panel A) evaluation in group A, B and C, and the System Usability Scale (SUS) evaluation in group A and B at T1 (Panel B). At panel C and D correlation between ATI and length (years) of education and classification according to International Standard Classification of Education.


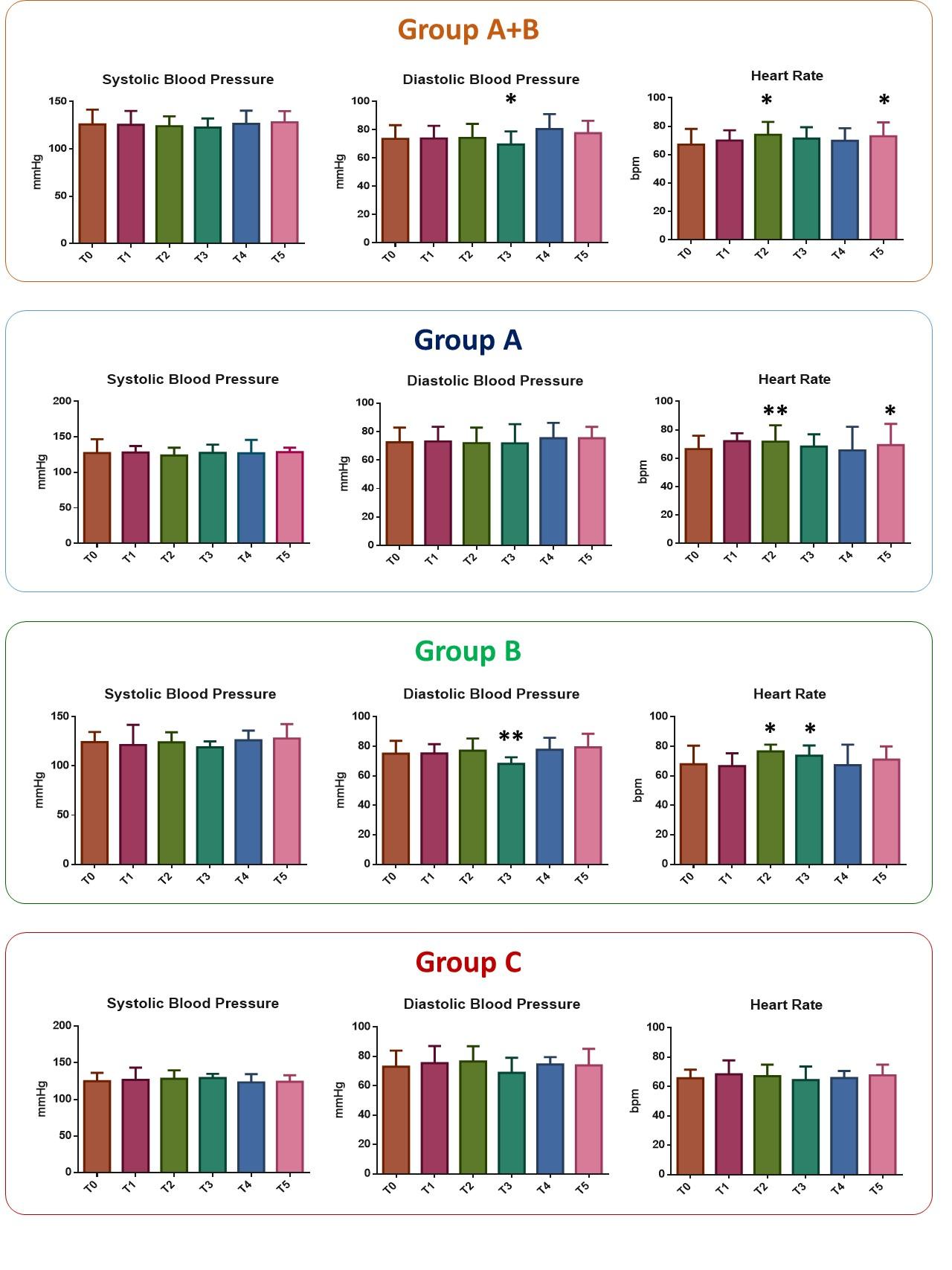


**Figure Supp 2**. Changes in Systolic blood pressure, Diastolic blood pressure and Heart rate in each group considering the different timepoint compared to baseline. * indicates p=0.04, ** indicates p = 0.01.

**Supplementary Table 1.** Comparison of comorbidities at baseline in each Groups A. B. and C.

|  | Group A | Group B | Group C | P |
| --- | --- | --- | --- | --- |
| Smokers (no/yes/former) | 20/4/9 | 20/3/10 | 16/6/9 | 0.553 |
| Hypertensive Heart Disease | 24 | 25 | 18 | 0.387 |
| Ischemic Heart Disease | 0 | 1 | 1 | 0.605 |
| Stroke | 1 | 1 | 2 | 0.789 |
| Hystory of Atrial Fibrillation | 1 | 2 | 1 | 0.761 |
| Hystory of Pulmonary Embolism | 0 | 0 | 0 | 0.998 |
| Arrhythmia | 3 | 6 | 2 | 0.258 |
| Diabetes | 5 | 5 | 5 | 0.998 |
| Dyslipidemia | 21 | 19 | 15 | 0.258 |
| COPD | 2 | 1 | 2 | 0.817 |
| History of cancer | 4 | 2 | 6 | 0.345 |
| Metabolic syndrome | 12 | 15 | 11 | 0.528 |
| PCOS | 0 | 1 | 1 | 0.605 |
| Chronic kidney disease | 3 | 5 | 3 | 0.648 |
| OSAS | 5 | 2 | 3 | 0.451 |

**Supplementary Table 2.** Comparison of drugs at baseline in each Groups A. B. and C.

|  | Group A | Group B | Group C | P (A vs B) | P (A vs C) | P (B vs C) |
| --- | --- | --- | --- | --- | --- | --- |
| number of medication assumed (median [IQR]) | 2 [1-3] | 2 [1-3] | 2 [1-3] | 0.841 | 0.275 | 0.316 |
| Angiotensin converting enzyme (ACE) inhibitors | 5 | 6 | 7 | 0.628 | 0.533 | 0.899 |
| Angiotensin receptor blockers (ARBs) | 17 | 17 | 12 | 0.715 | 0.231 | 0.124 |
| Thiazide | 9 | 9 | 8 | 0.827 | 0.784 | 0.627 |
| Indapamide | 3 | 0 | 2 | 0.093 | 0.648 | 0.175 |
| Beta-blockers | 10 | 9 | 9 | 0.968 | 0.792 | 0.827 |
| Calcium blockers channels | 12 | 10 | 7 | 0.795 | 0.184 | 0.298 |
| Statins | 16 | 12 | 17 | 0.503 | 0.814 | 0.369 |
| Ezetimibe | 3 | 3 | 4 | 0.910 | 0.695 | 0.788 |
| Acetylsalicylic acid | 6 | 7 | 6 | 0.987 | 0.934 | 0.931 |
| Antiplatelet | 2 | 2 | 2 | 0.875 | 0.965 | 0.841 |
| Direct oral anticoagulants | 1 | 2 | 0 | 0.654 | 0.333 | 0.198 |

**Supplementary Table 3.** Comparison of clinical and laboratory parameters between Groups A. B. and C.

|  | Group A | Group B | Group C | P (A vs B) | P (A vs C) | P (B vs C) |
| --- | --- | --- | --- | --- | --- | --- |
| Age (years) | 60.79 ± 13.12 | 62.12 ± 12.93 | 61.64 ± 13.88 | 0.718 | 0.827 | 0.899 |
| Sex (Male/Female) | 19/15 | 21/12 | 21/15 | 0.668 | 0.500 | 0.778 |
| Weight (Kg) | 81 ± 13.93 | 79.5 ± 17.35 | 80.3 ± 17.49 | 0.747 | 0.896 | 0.870 |
| Height (cm) | 166.5 ± 9.12 | 167.6 ± 7.47 | 165.6 ± 11.35 | 0.609 | 0.776 | 0.446 |
| Body Mass Index (Kg/m^2^) | 29.2 ± 4.33 | 28.3 ± 5.7 | 29.2 ± 4.46 | 0.533 | 0.997 | 0.547 |
| Neck circumference (cm) | 38.8 ± 3.07 | 38.5 ± 3.54 | 38.2 ± 4.08 | 0.755 | 0.597 | 0.800 |
| Abdomen circumference (cm) | 101.6 ± 9.68 | 98.9 ± 13.46 | 101.4 ± 13.6 | 0.406 | 0.945 | 0.521 |
| Systolic Blood Pressure (mmHg) | 128.85 ± 19.034 | 122.07 ± 24.67 | 124.73 ± 11.80 | 0.204 | 0.151 | 0.719 |
| Diastolic Blood Pressure (mmHg) | 74.78 ± 12.97 | 76.46 ± 10.97 | 74.09 ± 11.46 | 0.929 | 0.376 | 0.253 |
| Heart rate (bpm) | 68.63 ± 9.43 | 68.96 ± 9.42 | 65.27 ± 9.40 | 0.451 | 0.089 | 0.948 |
| Creatinine (mg/dl) | 0.94 ± 0.29 | 0.96 ± 0.30 | 0.88 ± 0.23 | 0.786 | 0.488 | 0.339 |
| Glomerular Filtration Rate (ml/min) | 80.83 ± 19.65 | 81.23 ± 21.38 | 86.15 ± 20.48 | 0.946 | 0.386 | 0.435 |
| Total Cholesterol (mg/dl) | 168.5 ± 34.73 | 174.28 ± 38.35 | 158.15 ± 31.28 | 0.593 | 0.318 | 0.136 |
| LDL Cholesterol (mg/dl) | 100.91 ± 34.18 | 109.96 ± 34.98 | 89.58 ± 27.40 | 0.380 | 0.246 | **0.040** |
| HDL Cholesterol (mg/dl) | 50.77 ± 12.42 | 52.29 ± 13.04 | 52.42 ± 16.32 | 0.688 | 0.716 | 0.977 |
| Triglycerides (mg/dl) | 104.18 ± 40.01 | 90.417 ± 28.39 | 103.72 ± 32.35 | 0.183 | 0.969 | 0.165 |
| Glucose (mg/dl) | 97.87 ± 24.92 | 92.88 ± 17.04 | 90.71 ± 11.82 | 0.419 | 0.280 | 0.651 |
| Urinary Protein to Creatinine Ratio | 133.09 ± 66.34 | 128.83 ± 134.17 | 120.67 ± 167.20 | 0.634 | 0.651 | 0.869 |
| Urinary Albumin to Creatinine Ratio | 20.43 ± 11.50 | 25.91 ± 85.85 | 7.50 ± 4.89 | 0.427 | 0.364 | 0.399 |

**Supplementary Table 4.** Comparison of echocardiographic parameters between patients in Groups A. B. and C.

|  | Group A | Group B | Group C | P (A vs B) | P (A vs C) | P (B vs C) |
| --- | --- | --- | --- | --- | --- | --- |
| Left Ventricle Ejection Fraction (%) | 61.79 ± 7.44 | 62.72 ± 3.55 | 61.31 ± 4.00 | 0.577 | 0.792 | 0.212 |
| Septal Interventricular Wall (mm) | 13.61 ± 1.50 | 12.92 ± 1.08 | 12.73 ± 0.99 | 0.077 | **0.031** | 0.545 |
| Posterior Wall diameter | 13.00 ± 1.57 | 12.68 ± 1.25 | 12.133 ± 1.48 | 0.451 | 0.780 | 0.211 |
| Internal Diameter of the Left Ventricle (mm) | 49.22 ± 5.13 | 47.57 ± 4.83 | 47.045 ± 4.53 | 0.267 | 0.152 | 0.719 |
| Left Ventricular Mass (g) | 271.16 ± 66.98 | 243.07 ± 55.27 | 224.89 ± 53.07 | 0.139 | **0.021** | 0.304 |
| Left Ventricular Mass Index (g/m^2^) | 141.77 ± 31.72 | 126.76 ± 30.26 | 118.50 ± 24.2 | 0.112 | **0.014** | 0.385 |
| Left Ventricular Mass Index^2.7^ (g/m^2.7^) | 69.04 ± 19.45 | 60.55 ± 14.45 | 59.05 ± 14.16 | 0.113 | 0.770 | 0.746 |
| RelativeWall Thickness | 0.53 ± 0.07 | 0.55 ± 0.07 | 0.52 ± 0.08 | 0.480 | 0.734 | 0.330 |
| E velocity (cm/s) | 69.55 ± 18.03 | 67.47 ± 15.94 | 66.33 ± 15.49 | 0.706 | 0.610 | 0.845 |
| A velocity (cm/s) | 83.26 ± 18.35 | 72.33 ± 13.21 | 78.83 ± 18.55 | **0.046** | 0.520 | 0.271 |
| e’ velocity (cm/s) | 7.31 ± 2.00 | 8.21 ± 2.20 | 8.07 ± 2.14 | 0.198 | 0.330 | 0.859 |
| E/e’ | 9.86 ± 3.47 | 8.42 ± 1.17 | 8.65 ± 1.86 | 0.094 | 0.274 | 0.676 |

**Supplementary Table 5.** Comparison of SF-36 domain at baseline and in each timepoint for Groups A. B. and C as median and inter quartile [IQR] range. Star (*) indicates a significant comparison (p<0.05) between the timepoint and T0.

|  | T0 | | | | | T1 | | | | T2 | | | | | | T3 | | | | | T4 | | | |
| --- | --- | --- | --- | --- | --- | --- | --- | --- | --- | --- | --- | --- | --- | --- | --- | --- | --- | --- | --- | --- | --- | --- | --- | --- |
|  | Gr. A | Gr. B | Gr. C | P ANOVA | Gr. A | | Gr. B | Gr. C | P ANOVA | | Gr. A | Gr. B | Gr. C | P ANOVA | Gr. A | | Gr. B | Gr. C | P ANOVA | Gr. A | | Gr. B | Gr. C | P ANOVA |
| Physical functioning | 95 [75-100] | 95 [80- 100] | 95 [80- 100] | 0.755 | 85 [66.25- 95]***** | | 85 [65- 95] | 90 [80- 95] | 0.197 | | 85 [55- 95] | 90 [56.25- 95] | 90 [72.50- 95] | 0.208 | 90 [62.5- 95] | | 90 [63.75- 95] | 90 [65- 95] | 0.942 | 90 [58.75- 100] | | 87.5 [75- 97.5] | 90 [70- 100] | 0.818 |
| Role limitation due to physical health | 100 [25- 100] | 100 [75- 100] | 100 [50- 100] | 0.515 | 100 [0- 100] | | 100 [25- 100] | 100 [25- 100] | 0.471 | | 50 [0- 100] | 100 [18.75- 100] | 100 [25- 100] | 0.501 | 100 [25- 100] | | 100 [25- 100] | 100 [25- 100] | 0.907 | 75 [12.5- 100] | | 75 [0- 75] | 75- 37.5- 87.5] | 0.631 |
| Role limitations due to emotionals problems | 100 [33-100] | 100 [66.67- 100] | 100 [33.33- 100] | 0.605 | 100 [33.33- 100] | | 100 [66.67- 100] | 100 [0- 100] | 0.104 | | 100 [33.33- 100] | 100 [33.33- 100] | 100 [0- 100] | 0.377 | 100 [33.33- 100] | | 100 [33.33- 100] | 100 [33.33- 100] | 0.569 | 83.33 [33.33- 100] | | 83.33 [41.67- 100] | 100 [0- 100] | 0.865 |
| Energy/fatigue | 50 [50-55] | 55 [50- 75] | 55 [50- 70] | 0.154 | 50 [50- 55] | | 55 [50- 65] | 50 [45- 65] | 0.258 | | 50 [35- 55] | 62.5 [45- 75] | 55 [40- 70] | 0.299 | 55 [40- 70] | | 60 [45- 76.25] | 55 [40- 70] | 0.628 | 57.5 [47.5- 63.75] | | 65 [60- 83.75] | 60 [52.5- 77.5] | 0.209 |
| Emotions well-being | 72 [64- 84] | 64 [64- 84] | 64 [52- 76] | 0.094 | 78 [55-84] | | 68 [64- 84] | 64 [40- 72] | **0.030** | | 72 [32- 84] | 76 [58- 85] | 56 [38- 80] | **0.002** | 72 [52- 84] | | 72 [56- 84] | 60 [44- 80] | **0.030** | 70 [54- 83] | | 72 [50- 80] | 56 [50- 78] | **0.030** |
| Social functioning | 75 [62.5- 100] | 62.5 [62.5- 87.5] | 62.5 [50- 87.5] | 0.182 | 68.75 [59.38- 100] | | 62.5 [62.5- 87.5] | 62.5 [50- 75] | 0.107 | | 75 [50- 100] | 75 [59.38- 90.63 | 75 [50- 93.75] | 0.567 | 75 [50- 87.5] | | 75 [62.5- 87.5] | 75 [50- 87.5] | 0.856 | 87.5 [53.13- 100] | | 75 [65.63- 87.50 | 75 [50- 93.75 | 0.569 |
| Pain | 100 [67.5- 100] | 100 [50- 100] | 90 [55- 100] | 0.171 | 95 [65-100] | | 90 [50- 100] | 80 [45- 100] | 0.447 | | 67.5 [45- 100] | 72.5 [45- 100] | 75 [32.5- 90] | 0.843 | 80 [57.5- 95] | | 80 [48.75- 90] | 77.50 [45- 90] | 0.910 | 78.75 [60- 100] | | 72.5 [59.38- 97.5 | 67.50 [45- 90] | 0.557 |
| General Health | 60 [45- 65] | 60 [50- 65] | 60 [55- 70] | 0.709 | 57.5 [45- 65] | | 60 [50- 75] | 60 [50- 70] | 0.770 | | 55 [40- 75] | 55 [45- 75] | 65 [45- 70] | 0.801 | 55 [45- 75] | | 55 [45- 71.25] | 55 [45- 70] | 0.901 | 55 [42.5- 72.5] | | 57.5 [41.25- 68.75 | 55 [40- 67.50] | 0.846 |

**Supplementary Table 6.** Comparison of MARS-5 evaluation for drug compliance at baseline and each timepoint for Groups A. B. and C as median and inter quartile [IQR] range.

|  | T0 | T1 | p vs T0 | T2 | p vs T0 | T3 | p vs T0 | T4 | p vs T0 | T5 | p vs T0 |
| --- | --- | --- | --- | --- | --- | --- | --- | --- | --- | --- | --- |
| **GROUP A+B** | 18 [17.75 -19] | 23 [22-23] | **0.001** | 23 [22-24] | **0.001** | 23 [23-24] | **0.001** | 23 [23-24] | **0.001** | 23 [22-24] | **0.001** |
| **GROUP A** | 18 [17.5-19] | 23 [23-24] | **0.001** | 23 [23-23] | **0.001** | 23 [22.5-24] | **0.001** | 23 [22.5-24] | **0.001** | 1.5 [0-2.75] | **0.001** |
| **GROUP B** | 18 [17.5-19] | 22 [22-23] | **0.001** | 23 [22-24] | **0.001** | 23 [23-24] | **0.001** | 23 [22-24] | **0.001** | 1 [0-3] | **0.001** |
| **GROUP C** | 18 [17.75-19] | 19 [18.75-20] | **0.010** | 18 [17.75-19] | 0.999 | 19 [18.75-20] | **0.020** | 19 [18.75-20] | **0.020** | 19 [18.75-19] | **0.040** |

**Supplementary Table 7.** Comparison of number of antihypertensive drugs at baseline and in each timepoint for Groups A. B. and C as median and inter quartile [IQR] range.

|  | T0 | T1 | p vs T0 | T2 | p vs T0 | T3 | p vs T0 | T4 | p vs T0 | T5 | p vs T0 |
| --- | --- | --- | --- | --- | --- | --- | --- | --- | --- | --- | --- |
| **GROUP A+B** | 2 [2-3] | 2 [1-3] | 0.999 | 2 [1-2,75] | **0.020** | 2 [0-3] | **0.010** | 1 [0-3] | **0.001** | 1 [0-3] | **0.001** |
| **GROUP A** | 2 [1-3] | 2 [1-3] | 0.999 | 2 [1-3] | 0.188 | 2 [0-2] | **0.040** | 1 [0-3] | **0.030** | 1.5 [0-2.75] | **0.010** |
| **GROUP B** | 2 [1-3] | 2 [1-3] | 0.999 | 2 [1-3] | 0.344 | 1,5 [0-3] | **0.080** | 1 [0-3] | **0.020** | 1 [0-3] | **0.020** |
| **GROUP C** | 2 [1-3] | 2 [1-3] | 0.999 | 2 [1-2,75] | 0.999 | 2 [1-3] | 0.999 | 2 [1-2,75] | 0.999 | 1.5 [1-2.25] | 0.999 |
